# The association between obstructive sleep apnea and total cerebral small vessel disease burden—Neurovascular insights of OSA

**DOI:** 10.3389/fnins.2026.1916857

**Published:** 2026-09-04

**Authors:** Rui Zhao, Guangze Cui, Xiaofei Zhang, Yinxia Bai, Zhiping Peng, Hongyi Zhao, Ruiqi Wang, Wenjuan Ma, Dongsheng Lv

**Affiliations:** 1 Department of Sleep Medicine, Inner Mongolia Autonomous Region Mental Health Center, Hohhot, China; 2 Hohhot Third Hospital (Hohhot Mental Health Center), Hohhot, China; 3 Department of Sleep Medicine, 984 Hospital of the Chinese People’s Liberation Army Joint Logistic Support Force, Beijing, China; 4 Division of Scientific Research, Inner Mongolia Autonomous Region Mental Health Center, Hohhot, China; 5 Department of Radiology, Inner Mongolia Autonomous Region Mental Health Center, Hohhot, China

**Keywords:** cognitive function, obstructive sleep apnea, polysomnography, apnea hypopnea index, cerebral bloodflow

## Abstract

**Objective:**

This study was conducted to assess the relationship between the severity of obstructive sleep apnea (OSA) and the total burden score of cerebral small vessel disease (CSVD), global cerebral blood flow (CBF), and cognitive function. Moreover, the internal pathway through which OSA induces cognitive impairment by affecting cerebral perfusion, and overall cerebral small vessel lesions was determined in this study.

**Methods:**

In total, 94 patients who received polysomnography (PSG) at the Mental Health Center of Inner Mongolia Autonomous Region from October 2024 to February 2026 were included in this study. Based on the apnea-hypopnea index (AHI), all individuals were classified into the control and the mild group (AHI < 15 times/h, *n* = 26), the moderate OSA group (15 ≤ AHI < 30 times/h, *n* = 27), and the severe OSA group (AHI ≥ 30 times/h, *n* = 41). Demographic information was collected from all patients, and their cognitive performance was evaluated using the Mini-Mental State Examination (MMSE), Trail Making Test (TMT), Choice Reaction Time task (CRT), Digit Symbol Substitution Task (DSST), and the Trails test with a paradigm similar to Part B of the Trail Making Test. Each patient underwent 3.0T magnetic resonance imaging, including conventional sequences, susceptibility weighted imaging (SWI), and arterial spin labeling (ASL). Finally, the total CSVD burden score was assessed.

**Results:**

The total CSVD burden and perivascular space (PVS) score in the severe OSA group were significantly higher than those in the moderate group and the control group (*P* < 0.05). The total CSVD burden was significantly positively correlated with the mean global CBF (*P* < 0.05). After the BMI was adjusted, the severity of OSA remained independently associated with the mean global CBF (*P* < 0.05). Total CSVD burden, PVS, lacunes, and global CBF were significantly associated with cognitive function domains (*P* < 0.05), which indicated that CSVD and global CBF abnormalities may jointly participate in OSA-related cognitive function.

**Conclusion:**

The severity of OSA might be related to the CSVD burden score. Neuroimaging characteristics strongly related to cognitive function in patients suffering from OSA, and abnormal global CBF may serve as a non-invasive imaging marker for evaluating neurocognitive damage.

## 1 Introduction

Obstructive sleep apnea (OSA) is a common disorder that occurs due to recurrent partial or complete blockage of the upper airway during sleep. OSA affects about 936 million people worldwide (Benjafield et al., 2019). It is characterized by repeated episodes of apnea or hypopnea, which in turn lead to intermittent hypoxia, hypercapnia, and disturbance of the sleep architecture. These pathophysiological alterations may induce multi-system damage, including hypertension, coronary heart disease, arrhythmias, type 2 diabetes mellitus, and cerebral small vessel disease (Arvanitakis et al., 2017; Blair et al., 2017; Fazekas et al., 1987; van Straaten et al., 2006).

Cerebral small vessel disease (CSVD) is a clinical, neuroimaging, and pathological syndrome caused by lesions involving the cerebral small perforating arteries, arterioles, capillaries, and venules with diameters of 40–200 μm. CSVD generally develops silently and gradually leads to cognitive dysfunction and other clinical symptoms (Wardlaw et al., 2013). It may also occur in an acute form, presenting as lacunar infarction, intracerebral hemorrhage, and subarachnoid hemorrhage, which substantially deteriorates the quality of life of patients and creates a considerable social burden. The severity of CSVD can be assessed using neuroimaging markers, including white matter hyperintensities (WMH), perivascular spaces (PVS), cerebral microbleeds (CMBs), and lacunes (Duering et al., 2023).

Previous clinical studies have reported a relationship between the severity of OSA and an increase in total CSVD burden (Chokesuwattanaskul et al., 2020; Huang et al., 2020; Lee et al., 2023; Zhou and Zhang, 2025). However, relevant meta-analyses have also revealed that the severity of OSA is independently associated with WMH, whereas findings on the association between OSA and other imaging markers, such as CMBs and lacunes, remain inconsistent across studies (Chokesuwattanaskul et al., 2020; Koo et al., 2017). The overall effect of CSVD can be evaluated by comprehensively assessing MRI imaging markers. For example, Staals et al. incorporated the four most representative imaging features of CSVD, including lacunes, WMH, CMBs, and PVS, to comprehensively evaluate the total CSVD burden (Saddler, 2004).

However, unambiguous and strong evidence is still lacking. Cerebral hypoperfusion and hyperperfusion were reported in recent studies on OSA (Xiao et al., 2022; Yan et al., 2021). Additionally, the imaging features of CSVD, global CBF, and the cognitive function of patients may be mutually associated. In recent studies, arterial spin labeling (ASL) was reported as a novel, strong, and non-invasive technique that reflected the function of the human brain and perfusion physiology (Petersen et al., 2006).

To summarize, in this study, we hypothesized that OSA increases the total CSVD burden and results in abnormal CBF. Using multimodal MRI that combined structural sequences with ASL, in this study, total CSVD burden, individual imaging markers, and global CBF were evaluated in OSA patients with different levels of disease severity. Based on cognitive function indicators, the intrinsic relationships among MRI features of increased CSVD burden, global CBF, and cognitive function were investigated in OSA patients to provide imaging evidence for the early clinical recognition of OSA-related brain injury.

## 2 Subjects and methods

### 2.1 Study participants

The participants included patients who visited the outpatient clinic of the Inner Mongolia Autonomous Region Mental Health Center between October 30, 2024, and February 1, 2026, and underwent PSG monitoring. The sample size formula using one-way ANOVA was *N* = σ^2^, with a two-sided significance level of α = 0.05 and power (1–β) = 0.8. The number of groups was *k* = 3. Consulting the table yielded the non-centralized parameter η≈ 12.65. As determined from previous studies (Mullins et al., 2021; Mullins et al., 2025), the estimated effect size f ≈ 0.365 was used, resulting in a total sample size of at least 94 cases for this study. Polysomnography (PSG) is the gold standard for OSA diagnosis. As per the International Classification of Sleep Disorders revised edition (ICSD-3-TR) published by the American Academy of Sleep Medicine (AASM) in 2023 and the Guidelines for the Diagnosis and Treatment of Adult Obstructive Sleep Apnea issued by the Chinese Thoracic Society (Chinese Thoracic Society and Chinese Medical Association, 2026), the diagnosis of OSA in adults requires the combination of clinical symptoms and objective detection evidence. OSA can be diagnosed when predominantly obstructive respiratory events, including obstructive apneas, mixed apneas, hypopneas, and respiratory effort-related arousals, occur at a rate of ≥ 15 events/h, or when predominantly obstructive respiratory events occur at a rate of ≥ 5 events/h, along with at least one typical clinical symptom, such as sleepiness, fatigue, insomnia, nocturnal gasping, witnessed snoring, or witnessed apneas. AHI is the principal index for classifying the severity of OSA: mild OSA is defined as an AHI of 5 to < 15 events/h, moderate OSA as an AHI of 15 to < 30 events/h, and severe OSA as an AHI of ≥ 30 events/h. Based on these severity grading criteria, all participants were divided into three groups: the severe OSA group (AHI ≥ 30 events/h), the moderate OSA group (15 ≤ AHI < 30 events/h), and the no-to-mild group (AHI < 15 events/h). All participants had to meet uniform baseline eligibility criteria: age between 18 and 75 years, with complete clinical data and PSG monitoring records available for the full analysis, and able to cooperate with subsequent MRI examinations and related assessments. Individuals were excluded if they had a history of cerebrovascular diseases, including large cerebral infarction, intracerebral hemorrhage, or severe intracranial vascular stenosis. Additionally, patients with diagnosed neurological disorders that may substantially influence global CBF and total CSVD burden, such as Parkinson’s disease, Alzheimer’s disease, and epilepsy, or those with severe systemic diseases, including heart failure, chronic obstructive pulmonary disease, and severe obesity (body mass index, BMI > 37.5 kg/m^2^), were excluded. Individuals who were administered continuous positive airway pressure (CPAP) treatment within 3 months before enrollment, as well as those with implanted metal devices, electronic instruments, or other contraindications for MRI scanning, were also excluded. Moreover, participants who were diagnosed with moderate or severe OSA were further excluded from the no-to-mild group. All participants were informed of the study procedures and voluntarily provided signed informed consent. They were grouped based on the AHI obtained from their PSG reports. The study was approved by the Ethics Committee of the Inner Mongolia Autonomous Region Mental Health Center [Ethical Approval No.:(2024) Ethical Review No. (117)]. The flow diagram was shown in Supplementary material 1.

### 2.2 Methods

#### 2.2.1 General demographic data collection

General demographic data, including sex, age, and educational level, were obtained in this study using a structured questionnaire.

#### 2.2.2 Polysomnography

The Compumedics Grael polysomnography system (Compumedics Limited, Australia) was used to perform polysomnography. The system comprised six electroencephalography channels, two electrooculography channels, two submental electromyography (EMG) channels, bilateral tibialis anterior EMG channels, two electrocardiography channels, an oronasal thermocouple sensor, a nasal pressure sensor, uncalibrated thoracic and abdominal inductance plethysmography bands, a piezoelectric snoring sensor, a Nonin finger pulse oximeter probe, a body position sensor, and synchronized video and audio recordings. While monitoring the patients, trained technicians at the sleep center ensured that the signal collected was adequate. Then, the PSG data were manually scored by a physician from the sleep center who had received standardized training, based on the AASM Manual for the Scoring of Sleep and Associated Events: Rules, Terminology, and Technical Specifications, Version 3.0 (Cui and Duan, 2024).

#### 2.2.3 Assessment of cognitive function

All participants participated in a set of standardized neuropsychological tests covering several cognitive domains. The Mini-Mental State Examination (MMSE) was conducted to evaluate global cognitive function and included 30 items across seven domains as follows: temporal and spatial orientation, immediate memory, calculation, delayed recall, language, executive function, and visuospatial skills. The higher the total score (range 1–30), the better the overall cognitive performance (Folstein et al., 1975; Jia et al., 2021).

The Trails Making Test Part A (TMT-A) and Part B (TMT-B) were implemented to assess attention, visual scanning and processing speed, executive function, cognitive flexibility, and task-switching ability, respectively. The completion time for each part was recorded, where a shorter time indicated better corresponding cognitive function (Llinàs-Reglà et al., 2017). The choice reaction time task (CRT) was performed to evaluate attention, and the mean reaction time was recorded. A shorter reaction time indicated better corresponding cognitive function. The digit symbol substitution task (DSST) was performed to assess psychomotor speed, attention, and executive function, and the number of correct responses was recorded. A higher number of correct answers indicated better corresponding cognitive function. Another Trails test using a paradigm similar to that of the TMT-B was conducted to assess executive function, and its completion time was recorded. A shorter time represented better corresponding cognitive function (Dalby et al., 2022; McIntyre et al., 2017; Zhu et al., 2024).

#### 2.2.4 Collection of MRI data

As the typical manifestations of CSVD do not occur separately, the total CSVD imaging burden score can be used to more comprehensively evaluate the overall effect of CSVD (Writing Group of the “China Expert Consensus on Diagnosis Treatment of Cerebral Small Vessel Diseases” by the Professional Committee of Cerebral Small Vessel Diseases and China Research Hospital Association, 2021), confirm the diagnosis, assess severity, and predict prognostic outcomes (Liu et al., 2023). The Staals scoring criteria are as follows: (1) presence of one or more lacunes (1 point); (2) presence of any CMBs (1 point); (3) presence of white matter lesions as extensive periventricular and deep white matter damage, or diffusely confluent WMH (1 point); (4) presence of > 10 enlarged perivascular spaces (1 point). Low, moderate, and high CSVD burdens are indicated by scores of 1, 2–3, and 4, respectively. The MR Cerebral Small Vessel Disease Intelligent Analysis System (uAI Discover CSVD, United Imaging Healthcare Co., Ltd.) is an MRI-based artificial intelligence analysis system that can comprehensively and quantitatively evaluate the imaging features of CSVD and assess the CSVD burden, thereby helping efficiently and reliably diagnose CSVD (Li and Xu, 2026). The segmentation was executed on the uAI research portal, based on the procedure described in previous studies (Zhang et al., 2024). The uAI Discover CSVD system can be used to automatically calculate the Staals score to determine the total CSVD burden in patients (Zeng et al., 2024). The manual contouring results of the above CSVD markers by two experienced radiologists blinded to the participants (Xiaobin Bai and Wenjuan Ma for Readers) were regarded as the ground truth. Several techniques are currently used to assess the NVU and cerebral perfusion levels. Among these techniques, ASL uses endogenously magnetically labeled blood as a tracer to quantitatively measure global CBF. ASL can identify cerebral perfusion abnormalities with high sensitivity and reveal structural damage and functional impairment of the NVU; hence, it has been widely used in clinical research (Wu et al., 2024; Xue et al., 2021).

Whole-brain neuroimaging data were obtained using a 3.0T Siemens Skyra MRI scanner equipped with a 16-channel head/neck coil. All scans were standardized and conducted by two board-certified radiologists (attending level or above). The patients were placed in the supine position and fitted with 3M foam earplugs to decrease scanner noise, while sponge pads were used for head fixation to decrease motion artifacts. Before scanning was performed, participants were informed about the procedure and instructed to remain motionless with their eyes closed, maintaining an awake resting state to ensure high-quality imaging. The raw imaging data were exported and stored in the DICOM format using DVD-R discs. Next, the uAI Discover CSVD system was used for automated quantitative assessment of CSVD imaging markers and estimation of the total CSVD burden (Zeng et al., 2024). The MRI parameters are summarized in Table 1.

**TABLE 1:** MRI sequence parameters.

Serials	TE (ms)	TR (ms)	Slice thickness (mm)	Slice gap (mm)	Slice per slab	FOV (cm^2^)	Matrix	FA
3D ASL	16.38	5,000	3.00	0	–	–	64 × 63	180°
DWI	65	4,180	5.50	0	–	–	192 × 192	180°
SWI	20	27	2.00	0	–	–	256 × 200	15°
3D TOF MRA	3.43	20	0.60	0	–	–	384 × 216	18°
3D T1 MPRAGE	2.26	2,400	1.00	0	192	24 × 24	256 × 256	8°
T1 TSE dark-fluid	9	2,000	5.50	0	–	–	320 × 182	150°
T2 TIRM dark-fluid	90	8,600	5.50	0	–	–	320 × 208	120°
T2 TSE	105	3,790	5.50	0	–	–	384 × 250	130°

TE, echo time; TR, repetition time; FOV, field of view; FA, flip angle.

#### 2.2.5 Processing of MRI data

The MRI data were processed on the MATLAB R2022b platform using the SPM12 software along with EXPLOREASL GUI (version 0.5.0). The workflow is described below. (1) Data import and format conversion: The original DICOM images obtained from the MRI scans were imported and converted using the “DICOM to NIfTI” module in EXPLOREASL GUI. This process generated NIfTI files (nii/. nii. gz) that were compatible with subsequent analyses, while retaining essential scanning parameters (e.g., TR, TE, TI, and flip angle) within the image headers.

(2) Motion correction: Using the “Motion Correction” module in EXPLOREASL GUI, rigid-body registration (six degrees of freedom: translations along the x, y, and z axes; rotations of pitch, yaw, and roll) was performed on the ASL time series, with the first volume used as the reference. Motion parameters were calculated at each time point, with acceptable limits defined as translation < 0.5 mm and rotation < 0.5. Volumes exceeding these motion thresholds were removed, and interpolation correction was performed on the remaining volumes to generate motion-corrected standardized NIfTI data. This procedure ensured that subsequent CBF calculations remained unaffected by head motion artifacts.

(3) Arterial input function (AIF) extraction: Using an automated algorithm, the middle cerebral artery (MCA) region was located, followed by manual refinement of the boundaries of the region of interest (ROI). Then, the AIF curves were extracted, and their morphological quality was controlled.

(4) Fitting of the CBF model: A PASL quantification model was selected, and physiological parameters were configured to fit and generate voxel-wise relative CBF maps.

(5) Spatial normalization: The CBF maps were registered to the MNI152 standard brain template and resampled to a voxel size of 3 × 3 × 3 mm^3^ to obtain spatial consistency.

(6) Spatial smoothing: The CBF maps were smoothed using a Gaussian kernel with a 6-mm full width at half maximum (FWHM) to decrease spatial noise and increase the signal-to-noise ratio (SNR).

(7) Regional mean extraction: The mean CBF values of the predefined brain regions were extracted and exported as a structured data table based on the Anatomical Automatic Labeling (AAL) atlas.

(8) Quality control and outlier screening: The distributions of the CBF values and overall image quality were reviewed. Datasets with values beyond the physiological range, abnormal AIF profiles, or excessive registration errors were excluded to ensure that the results were reliable.

### 2.3 Statistical analysis

All statistical analyses were performed using the SPSS 25.0 statistical software. Categorical variables were expressed as frequencies [n (%)], and intergroup comparisons were made by conducting the Chi-square (χ^2^) test, with the phi coefficient (φ) reported as the effect size. The Kolmogorov-Smirnov and Shapiro-Wilk tests were performed to evaluate the normality of continuous variables, and Levene’s test was conducted to assess variance homogeneity. Continuous variables that did not satisfy the normal distribution assumption were described as the median [*M* (P25, P75)]. To determine differences between groups, the Kruskal-Wallis H test was conducted, followed by Dunn’s *post-hoc* test with Bonferroni correction for pairwise comparisons. The effect size was indicated by the epsilon coefficient (ε).

All regression models were uniformly adjusted for age, sex, body mass index, hypertension, and diabetes. Educational qualification was set as dummy variables, with junior high school education as the reference group, and senior high school, college, and higher levels of education as grouped dummy variables. The global mean CBF had a right-skewed distribution. Sensitivity analyses were conducted by constructing models using both the raw CBF mean values and natural logarithm-transformed ln_CBF values. As the positive rates of lacunar infarcts and WMH were relatively low, model convergence was unstable, and thus these indicators were not included in the analyses.

First, single-exposure independent effect analyses were conducted. Regression models were constructed with the OSA severity grade and cerebral blood flow as core exposures, respectively. Ordinal logistic regression was performed with the total CSVD burden as the ordinal outcome, and the parallel lines test was simultaneously conducted to assess the proportional odds assumption. Binary logistic regression was performed with severe PVS and CMBs as binary outcomes, accompanied by the Hosmer-Lemeshow goodness-of-fit test. Multiple linear regression was performed, with CBF regarded as the continuous outcome. All regressions forced all confounding variables into the models using the Enter method.

To distinguish the independent effects of OSA severity grading and cerebral blood flow on CSVD, joint adjusted models were further established, in which the OSA rank and CBF indicators were simultaneously incorporated into the same equation to adjust for mutual confounding between the two factors. Ordinal logistic regression regarded the total CSVD burden as the outcome, binary logistic regression regarded severe PVS as the outcome, and sensitivity was verified with ln_CBF.

The Benjamini–Hochberg method was used to conduct FDR multiple comparison correction for eight groups of baseline single-exposure independent association tests to control Type I error. The joint adjusted models and sensitivity verification models belonged to stratified internal validation analyses and did not undergo multiple comparison correction. Decisions were made based on original P-values. The results of ordinal logistic and binary logistic regression were described by adjusted odds ratios (ORs) and 95% confidence intervals (CIs). The results of multiple linear regression were presented using the unstandardized regression coefficient B, the standardized coefficient β, and 95% CIs. All differences were considered to be statistically significant at *P* < 0.05.

## 3 Results

### 3.1 Comparison of demographic characteristics

The differences in the demographic characteristics, including sex ratio, age, and years of education, were not statistically significant among the three groups, suggesting that the baseline characteristics were comparable. However, BMI was significantly different among the three groups (*P* < 0.05). *Post-hoc* pairwise comparisons indicated that BMI in the severe group was significantly different from that in the no-to-mild and moderate groups (*P* < 0.05), whereas no significant difference was detected between the no-to-mild and moderate groups (Table 2).

**TABLE 2:** Demographic characteristics of the three groups.

Group	Sex [*n*(%)]		Age [*M* (P25, P75), years]	BMI [*M*(P25, P75), kg/m^2^]	Years of education [*M* (P25, P75), years]
	Male	Female			
No to mild group (*n* = 26)	11 (42.31)	15 (57.69)	47.50 (35.00, 62.25)	23.48 (20.20, 26.09)	12.00 (9.00, 15.00)
Moderate group (*n* = 27)	12 (44.44)	15 (55.56)	64.00 (54.00, 66.20)	23.99 (22.19, 25.39)	9.00 (9.00, 12.00)
Severe group (*n* = 41)	24 (58.54)	17 (41.46)	54.00 (43.00, 61.00)	26.67(23.63, 28.88)	12.00 (9.00, 15.00)
φ/ε	0.151		0.024	0.042	0.010
χ^2^/H	2.144		5.077	15.427	0.980
P	0.342		0.079	< 0.001	0.613

BMI, body mass index.

### 3.2 Comparison of total CSVD burden and global CBF among the groups

Comparison of the total CSVD burden and global CBF across the three groups showed a significant difference in the total CSVD burden (*P* < 0.05). *Post-hoc* pairwise comparisons revealed that the total CSVD burden was significantly greater in the severe group than in the moderate group (*P* < 0.05), whereas the difference between the no-to-mild and moderate groups was not significant. The differences in the mean global CBF or the median global CBF among the three groups were not significant (Table 3).

**TABLE 3:** Total CSVD burden and global CBF among the three groups.

Group	Total CSVD burden [M(P25, P75), score]	CBF [*M*(P25, P75), mL/100 g/min]	
		CBF_Mean	CBF_Med
No to mild group (*n* = 26)	0.00 (0.00, 1.00)	55.05 (48.34, 62.51)	38.38 (29.83, 44.81)
Moderate group (*n* = 27)	0.00 (0.00, 1.00)	55.79 (49.06, 64.42)	37.37 (33.73, 48.10)
Severe group (*n* = 41)	1.00 (1.00, 1.00)	59.38 (53.55, 67.98)	37.02 (33.73, 41.52)
ε	0.030	0.019	0.018
H	8.068	3.083	2.843
P	0.018	0.214	0.241

CBF_Mean, mean global CBF; CBF_Med, median global CBF.

### 3.3 Comparison of CSVD imaging markers among the groups

By comparing the CSVD imaging markers among the three groups, a significant difference was found in the PVS scores (*P* < 0.05). *Post-hoc* pairwise comparisons showed that the PVS score was significantly higher in the severe group than in the moderate and no-to-mild groups (both *P* < 0.05), whereas the difference between the no-to-mild and moderate groups was not significant (Table 4). These findings indicated that total CSVD burden was mainly driven by PVS.

**TABLE 4:** CSVD imaging markers among the three groups.

Group	CSVD imaging markers [*M* (P25, P75), score]			
	WMH	lacunes	PVS	CMBs
No to mild group (*n* = 26)	0.00 (0.00, 0.00)	0.00 (0.00, 1.00)	0.00 (0.00, 0.00)	0.00 (0.00, 0.00)
Moderate group (*n* = 27)	0.00 (0.00, 0.00)	1.00 (0.00, 1.00)	0.00 (0.00, 0.00)	0.00 (0.00, 0.00)
Severe group (*n* = 41)	0.00 (0.00, 0.00)	0.00 (0.00, 1.00)	1.00 (0.00, 1.00)	0.00 (0.00, 1.00)
ε	0.000	0.029	0.090	0.026
*H*	0.000	2.667	8.416	2.459
*P*	1.000	0.263	0.015	0.292

WMH, white matter hyperintensities; lacunes, lacunar infarcts; PVS, perivascular spaces; CMBs, cerebral microbleeds.

### 3.4 Correlation between total CSVD burden and global CBF

**FIGURE 1:**
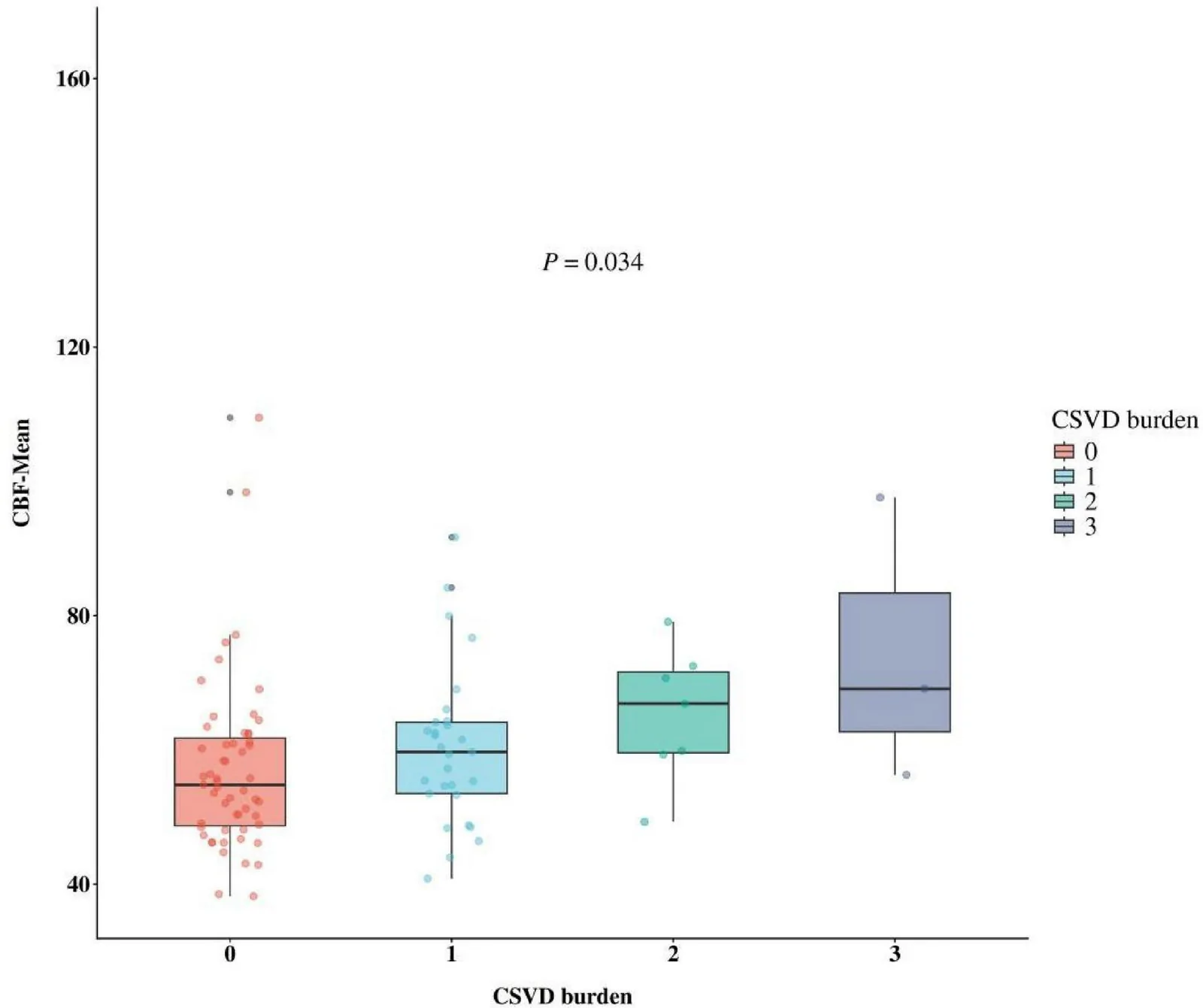
Correlation between total CSVD burden and global CBF.

The results of the correlation analysis showed that total CSVD burden was significantly and positively correlated with the mean global CBF (*P* < 0.05), which indicated that cerebral blood perfusion increased along with an increase in total CSVD burden (Figure 1).

### 3.5 Relationship between global CBF and individual CSVD imaging markers

The relationship between OSA severity grading, cerebral blood flow, and total CSVD burden was demonstrated by a single-exposure ordered logistic regression (Table 5). After adjusting for confounding factors, a high OSA severity grade and a high mean whole-brain cerebral blood flow were both found to serve as independent risk factors for a greater CSVD burden, with differences remaining statistically significant after FDR correction (all adjusted *P* = 0.033). Sensitivity testing via ln_CBF yielded consistent results with the original CBF analysis, which confirmed that the findings were robust.

**TABLE 5:** Ordinal logistic regression analysis of OSA classification, CBF, and the total CSVD burden.

Analysis type	Independent variable	OR calibrate	95%CI	Original *p*-value	FDR-corrected *p*-value
Independent analysis of single exposure	OSArank	1.735	1.024∼2.949	0.011	0.033
	CBFmean	1.042	1.009∼1.072	0.011	0.033
	ln_CBF	16.310	–	0.008	–
Joint calibration model	OSArank^1^	1.735	1.024∼2.949	0.041	–
	CBFmean^1^	1.035	1.002∼1.068	0.037	–
	OSArank^2^	1.887	1.117∼3.165	0.016	–

All models were adjusted for age, sex, BMI, hypertension, diabetes, and educational qualification. The *P*-values from the proportional odds test for ordered logistic regression were all > 0.05, satisfying the proportional odds assumption.

^1^ indicates that OSArank and CBFmean were simultaneously included in the model and mutually adjusted.

^2^ on model. FDR correction was only performed on independent associations of single baseline exposures. Joint adjustment models and sensitivity models were not subject to correction. All results were considered to be statistically significant at *P* < 0.05.

Additionally, incorporating both OSA severity scores and CBF into a joint calibration model revealed that after mutual adjustment, both remained independent predictors of total CSVD burden (OSArank: OR = 1.735, *P* = 0.041; CBFmean: OR = 1.035, *P* = 0.037), indicating that OSA and cerebral blood flow contribute to the CSVD burden through two distinct pathways. The results were consistent when the ln-CBF sensitivity joint model was implemented. All parallel line tests for ordered logistic regression yielded *P*-values > 0.05, satisfying the proportional hazards assumption.

### 3.6 The relationship between OSA grading, CBF, and PVS

The results of the univariate binary logistic regression analysis (Table 6) revealed that after adjusting for confounding factors, higher OSA severity grading significantly increased the risk of severe PVS, and the difference remained significant following FDR correction (adjusted *P* = 0.046). Neither raw cerebral blood flow nor log-transformed cerebral blood flow was significantly associated with severe PVS.

**TABLE 6:** Binary logistic regression analysis of OSA grading, CBF, and severe PVS.

Analysis type	Independent variable	OR calibrate	95%CI	Original *p*-value	FDR-corrected *p*-value
Independent analysis of single exposure	OSArank	2.677	1.096∼6.539	0.023	0.046
	CBFmean	1.024	0.982∼1.067	0.265	0.265
	ln_CBF	6.800	0.396∼116.736	0.186	–
Joint calibration model	OSArank^1^	2.677	1.096∼6.539	0.031	–
	CBFmean^1^	1.018	0.976∼1.062	0.403	–
	OSArank^2^	2.648	1.082∼6.484	0.033	–
	ln_CBF^2^	4.655	0.259∼83.826	0.297	–

All models were adjusted for age, sex, BMI, hypertension, diabetes mellitus, and educational qualification. The Hosmer-Lemeshow test yielded *P* > 0.05 for all binary logistic regression models, indicating good model fit.

^1^: OSArank and CBFmean were simultaneously included in the equation with mutual adjustment.

^2^: OSArank and ln_CBF were simultaneously included, forming the sensitivity validation model. FDR correction was only performed on independent associations of single baseline exposures; joint adjustment models and sensitivity models did not undergo FDR correction. All results were considered to be statistically significant at *P* < 0.05.

In the jointly adjusted model, OSA grading remained an independent risk factor for severe PVS after adjusting for cerebral blood flow (OR = 2.677, *P* = 0.031), whereas cerebral blood flow was not statistically significant after adjusting for OSA (*P* = 0.403). These findings indicated that the effect of OSA on severe PVS was independent of alterations in global cerebral perfusion levels. Similar results were obtained in the sensitivity validation using ln_CBF. The *P*-values of the Hosmer-Lemeshow test for all binary logistic regression models were > 0.05, suggesting good model fit.

### 3.7 Relationship between OSA classification and cerebral microhemorrhage

The results of single-exposure binary logistic regression revealed that, after adjusting for confounding factors, only a positive marginal trend was found between severe OSA and cerebral microhemorrhage, and the difference was not significant (OR = 1.702; original *P* = 0.091; FDR-adjusted *P* = 0.1092).

### 3.8 Relationship between severe grading of OSA and global mean cerebral blood flow

Hierarchical multiple linear regression was performed to analyze the independent effect of the severity of OSA on CBF. The mean CBF value was set as the dependent variable, and independent variables were entered stepwise in four blocks based on theoretical hypotheses. Model 1 included demographic variables (age, gender, and educational level); Model 2 added BMI; Model 3 also incorporated chronic disease variables (hypertension and diabetes); Model 4 added the OSA severity classification. The enter method was implemented with the significance level α = 0.05. All statistical analyses were performed using the SPSS software. VIF and tolerance were applied to test multicollinearity, and the Durbin-Watson test was conducted to detect residual autocorrelation.

The results of hierarchical regression analysis indicated that demographic variables in Model 1 significantly explained 18.4% of the variance in CBF (*F* = 5.019, *p* = 0.001). Older age (β = 0.250, *p* = 0.032) and male gender (β = −0.209, *p* = 0.039) were associated with higher CBF. After sequentially adding BMI (Δ*R*^2^ = 0.016, *p* = 0.184), hypertension, and diabetes (Δ*R*^2^ = 0.004, *p* = 0.796) based on Model 1, the explanatory power of the model did not improve significantly.

After adjusting for demographic factors, BMI, and chronic diseases, the OSA severity classification in Model 4 showed a marginally significant positive predictive effect on CBF (*B* = 2.92, 95% CI: −0.38 to 6.22, β = 0.189, *p* = 0.082). Each one-grade increase in OSA classification corresponded to an average increase of 2.92 units in the mean CBF. Gender remained an independent influencing factor of CBF (*B* = −6.74, 95% CI: −11.97 to −1.52, β = −0.264, *p* = 0.012), and females had significantly lower CBF than males. The final model explained 23.3% of the variance in CBF (adjusted *R*^2^ = 0.160). Collinearity diagnostics revealed that the VIF values of all independent variables were < 2, and the Durbin-Watson statistic was 1.850, which satisfied all model assumptions.

### 3.9 Correlation among cognitive test scores, CSVD imaging markers, and global CBF

Correlation analysis of the cognitive test scores, CSVD imaging markers, and global CBF showed that the total CSVD burden was significantly and positively correlated with the CRT results (*P* < 0.01), and negatively correlated with the DSST results (*P* = 0.014). Lacunes were significantly and negatively associated with the MMSE scores (*P* = 0.013). PVS was significantly and positively correlated with the CRT results (*P* < 0.01), and negatively correlated with the DSST results (*P* = 0.008). Mean global CBF showed significant positive correlations with the TMT-A and CRT results (*P* = 0.021 and 0.012), and a negative correlation with the DSST results (*P* = 0.045). These findings indicate that neuroimaging characteristics, including total CSVD burden, lacunes, and PVS, are important factors influencing cognitive function, and that global CBF is also associated with cognition (Table 7). No close relationship was found between CSVD severity/CBF and other hypoxic burden metrics, such as ODI3%, ODI4%, SpO_2_ Mean, SpO_2_ Nadir, T90, and RAI. The details are provided in Supplementary material 2.

**TABLE 7:** Correlation between cognitive function and MRI findings.

Variable	Total CSVD burden	Lacunes	PVS	CMBs	CBF_mean
MMSE	−0.181	−0.290#	−0.137	−0.143	−0.149
TMT-A	0.211	0.125	0.220	0.109	0.266#
TMT-B	0.146	−0.055	0.141	0.123	0.191
CRT	0.356#	0.134	0.435#	0.138	0.286#
DSST	−0.282#	−0.137	−0.304#	−0.156	−0.233#

#*P* < 0.05.

## 4 Discussion

In this study, controls with an AHI of < 15 events/h and patients with moderate-to-severe OSA were included. By comparatively examining AHI-, CSVD-, global CBF-, and cognitive function-related indices across the three groups, the associations between the severity of OSA and cerebral microvascular pathology, cerebral blood perfusion, and cognitive performance were investigated. The results revealed that patients with severe OSA had a significantly higher BMI than those in the moderate OSA group and control group. Total CSVD burden was positively associated with mean global CBF. After adjusting for BMI, the severity of OSA remained independently and positively associated with the mean global CBF, whereas no significant differences were detected in the core cognitive indices among the three groups. These findings provide clinical evidence for a better understanding of the pathophysiological mechanisms underlying the cerebral microvascular pathology related to OSA, and offer a useful reference for early intervention and protecting the brain function of patients with OSA.

### 4.1 Association between BMI and the severity of OSA

No significant differences in sex, age, or years of education were found among the control, moderate OSA, and severe OSA groups, which indicated that the baseline characteristics were comparable among the groups. However, the distribution of BMI across the three groups was significantly different. *Post-hoc* pairwise comparisons revealed that BMI in the severe OSA group was significantly higher than that in the control and moderate OSA groups. This clinical feature is consistent with previous findings on the relationship between OSA and BMI, suggesting that high BMI is an important correlate of OSA worsening and is particularly closely associated with severe OSA. Excessive fat deposition in the neck of obese patients may directly narrow the upper airway. Simultaneously, high intra-abdominal pressure further limits lung volume and weakens the stability of the airway. These factors make the upper airway more collapsible, thereby promoting the progression of OSA from the mild stage to the moderate-to-severe stage (Xu et al., 2026). Previous epidemiological studies have shown that obesity, especially central obesity, serves as a major risk factor for the onset and progression of OSA. The prevalence of OSA can reach 41% in patients with a BMI > 28 kg/m^2^, and it increases sharply to 78% in obese patients undergoing bariatric surgery. Moreover, a significant dose-response relationship exists between change in weight and the severity of OSA (Mukminin et al., 2026). The Wisconsin Sleep Cohort Study showed that a 10% weight gain over 4 years led to a 32% increase in the AHI and a six-fold increase in the risk of progression to moderate-to-severe OSA. Similarly, the Sleep Heart Health Study confirmed that a 10-kg weight gain over 5 years increased the probability of a 15 events/h increase in the AHI by 5.2 times in men and 2.5 times in women. These findings support the close relationship between weight gain and the aggravation of OSA. No significant difference in the BMI was found between the moderate OSA group and the control group, whereas the BMI increased significantly in patients with severe OSA. This suggests that BMI may serve as an aggravating factor for the severity of OSA rather than an initial pathogenic factor for moderate OSA. Other studies have reported that upper airway anatomical abnormalities form the core anatomical basis of OSA, which may independently cause moderate OSA even within the normal BMI range. As the severity of obesity increases, the accumulation of cervical fat further compresses the upper airway, which, along with underlying anatomical narrowing, promotes disease progression to a severe stage (Garvey et al., 2015).

### 4.2 Severity-stratified pattern of total CSVD burden in patients with OSA

In this study, a significant difference in total CSVD burden was found among the three groups. The severe OSA group showed a significantly higher total CSVD burden than the moderate group, whereas the difference between the moderate OSA and control groups was not significant. These findings are partly consistent with the conclusions of previous systematic reviews and meta-analyses. For example, a meta-analysis by Huang et al. (2020) found a positive association between moderate-to-severe OSA and core CSVD imaging markers such as WMH, which suggested that OSA may promote the accumulation of cerebral small vessel injury through pathophysiological processes, including intermittent hypoxia, endothelial dysfunction, and inflammatory responses (Huang et al., 2020). By further classifying OSA into moderate and severe groups, a significant difference in total CSVD burden was found only between the severe and moderate OSA groups; however, the difference between the moderate OSA group and the control group was not significant. This indicates a “threshold effect” in the increase in total CSVD burden: only when OSA advances to the severe stage does the accumulation of cerebral small vessel damage reach a detectable and significant level, whereas the intensity of pathological exposure in moderate OSA may be insufficient to produce a significant difference compared to the control group.

In this study, only the PVS burden differed significantly among the three groups, with significantly higher values in the severe OSA group than in the moderate group, and no significant difference between the moderate OSA and control groups. In contrast, WMH, lacunes, and CMBs did not differ significantly among the three groups. These findings are highly consistent with the conclusions of a large-sample prospective study by Lin et al. (2024) published in Radiology. In that study, patients with severe OSA had significantly higher PVS volume fractions and extracellular free water than those with mild OSA and controls, and PVS diffusion metrics were associated with cognitive impairment. These results indicate that PVS is a sensitive early biomarker of OSA-related damage to cerebral small vessels. Nocturnal hypoxia-reoxygenation in OSA causes fluctuations in cerebral blood flow and glymphatic clearance dysfunction. As a key anatomical component of the glymphatic system, the PVS is more vulnerable to acute injury. Therefore, differences between severe and moderate OSA appear earlier, whereas the difference between moderate OSA and controls does not reach statistical significance. However, this finding is inconsistent with those of the meta-analysis by Huang et al. (2020) in which no association was found between OSA and PVS. This discrepancy may be explained by the small sample sizes and high heterogeneity of PVS-related studies.

In this study, WMH, lacunes, and CMBs showed no significant differences among the groups, which differed from the conclusion by Huang et al. (2020) who found a positive correlation between moderate-to-severe OSA and WMH. This discrepancy probably occurred because these CSVD subtypes depend more strongly on chronic risk factors, such as long-term hypertension and aging, whereas most patients enrolled in our study were newly diagnosed with OSA, meaning that their disease duration and intensity of exposure may have been insufficient to induce significant differences. Moreover, meta-analyses in previous studies mainly compared patients with moderate-to-severe OSA with healthy controls, whereas this study focused on internal stratification within the moderate-to-severe OSA spectrum, which may more easily reveal differences in PVS, a marker that is highly sensitive to acute injury. Moreover, the finding in this study is consistent with the conclusion drawn by Huang et al. (2020) who showed that OSA is not associated with CMBs, collectively suggesting that the effect of OSA on different subtypes of CSVD shows heterogeneity.

A longitudinal study by Sommer et al. (2025) published in Neurology further supports the clinical value of PVS as a therapeutic target. The study showed that regular CPAP therapy stabilized basal ganglia PVS volume in patients with moderate-to-severe OSA, whereas poor adherence was associated with a significant increase in PVS volume. This protective effect was stronger among patients with more severe hypoxia and higher AHI, suggesting that OSA-related PVS damage may be reversible.

The findings of this study showed that the PVS burden was significantly higher in the severe OSA group than in the moderate group. This indicates that PVS serves both as a sensitive early biomarker of OSA-related cerebral small vessel damage and as an important potential target whose progression may be delayed by CPAP intervention. Moreover, the absence of a significant difference in PVS burden between the moderate OSA group and the controls suggests that starting standardized CPAP therapy at this stage may be more effective at preventing PVS progression, thereby providing evidence based on neuroimaging for early intervention in OSA patients with concomitant CSVD.

### 4.3 Correlation between mean global CBF and total CSVD burden in OSA patients

This study showed that greater OSA severity and higher global cerebral perfusion constitute two independent, mutually non-interfering risk factors for high total CSVD burden. After adjusting for confounders, including age, sex, BMI, hypertension, and diabetes mellitus, only a positive marginal trend with no statistical significance was identified between OSA severity grade and mean global cerebral blood flow. These observations are consistent with the core findings from multiple previous investigations addressing cerebral perfusion and metabolic alterations associated with OSA.

The neurovascular unit (NVU) serves as the core structural basis of cerebral microvascular homeostasis and comprises neurons, the blood-brain barrier, microglia, and the extracellular matrix that maintains the integrity of brain tissue (del Zoppo, 2010; Muoio et al., 2014; Zhào et al., 2018). CBF is widely regarded as a reliable quantitative indicator for assessing the functional impairment of NVU under chronic hypoperfusion (Ru et al., 2024; Zhou et al., 2022). A classical pathophysiological hypothesis was proposed in 2015 to explain the pathway between OSA and high total CSVD burden: chronic intermittent hypoxia and sleep architecture disruption in OSA patients may increase total CSVD burden by inducing ischemic-hypoxic injury to the NVU (Chen et al., 2017). Subsequent clinical observational studies preliminarily support this mechanistic model (Kerner and Roose, 2016).

From the perspective of acute hemodynamic fluctuation, obstructive respiratory events induce immediate cerebral circulatory disturbances. In a study involving patients with severe OSA conducted in 2023, Gregori-Pla et al. showed that obstructive respiratory events can trigger marked biphasic fluctuations in cerebral microcirculatory blood flow and oxygenation, and the magnitude of changes in CBF is positively correlated with the duration of the event and severity of apnea (Gregori-Pla et al., 2023). By conducting simultaneous sleep MRI, Dennison et al. demonstrated that during spontaneous apneic episodes in natural sleep, patients with OSA show significantly higher CBF, lower arterial oxygenation, and lower cerebral metabolic rate of oxygen, reflecting a clear state of neurovascular-metabolic uncoupling (Dennison et al., 2025). The high global CBF observed in the awake resting state in this study suggests a persistent daytime hemodynamic abnormality, which may represent the cumulative effect of recurrent nocturnal hyperperfusion and metabolic mismatch in patients with OSA.

From the perspective of chronic cumulative changes, long-term repeated hemodynamic disturbances may develop into compensatory pathological alterations and contribute to the progression of CSVD. Xiao et al. (2022) found a compensatory increase in CBF in brain regions such as the prefrontal cortex and precentral gyrus in OSA patients, accompanied by atrophy of the gray matter, suggesting that cerebral hyperperfusion may be a compensatory response to chronic neuronal injury. Consistent with this finding, the mean global CBF in our OSA cohort also showed a compensatory increase, further supporting the diffuse nature of hemodynamic compensation associated with OSA. This study also found that hyperperfusion was significantly correlated with CSVD markers (e.g., PVS and CMBs), providing observational evidence that abnormality in global CBF is closely associated with the progression of CSVD. Troudi Habibi et al. also explained that early cerebral hemodynamic abnormalities can be captured non-invasively by ASL and identified a core pathological pathway. Briefly, chronic intermittent hypoxia leads to cerebrovascular regulatory dysfunction, vascular remodeling, and disruption of the blood-brain barrier, which may eventually result in pathological hyperperfusion (Troudi Habibi et al., 2025).

After controlling for the confounding effect of BMI, we found that OSA was independently associated with elevated global CBF and higher CSVD burden. This finding supports that CBF can serve as a candidate and non-invasive biomarker for reflecting OSA-related neurovascular injury and determining the risk of CSVD.

Some studies have, however, reported inconsistent findings. Yadav et al. found a significant reduction in regional CBF within the principal sensorimotor fiber systems and motor regulation regions in OSA patients, with a lateralized distribution pattern (Yadav et al., 2013), which is different from our finding of high mean global CBF. This inconsistency occurred probably because of two reasons. First, Yadav et al. focused on alterations in regional CBF, whereas this study used mean global CBF, which is more sensitive for capturing diffuse compensatory hyperperfusion. Therefore, focal hypoperfusion may be masked by the overall global hyperperfusion state. Second, the study by Yadav et al. had a small sample size and lacked proper stratification of OSA severity, whereas our cohort mainly included patients with moderate-to-severe OSA, which more readily revealed the global compensatory response associated with chronic severe hypoxia.

Our findings showed associations among OSA severity, total CSVD burden, and global CBF, which may have some clinical implications. First, treatment of OSA might be an effective way to regress CSVD. Continuous positive airway pressure, the first choice against OSA, was found to decrease the effects of sleep apnea on trajectories of MRI-visible PVS volumes (Sommer et al., 2025). Second, CSVD may serve as the main mediator of OSA-related cognitive dysfunction (Ke et al., 2025). As this cross-sectional study could not establish a causal relationship, longitudinal studies are required to determine these possibilities.

### 4.4 Total CSVD burden and cerebral blood perfusion mediate OSA-related cognitive impairment

In this study, total CSVD burden was significantly and positively correlated with TMT-A and CRT results and negatively correlated with DSST results. Lacunes were significantly and negatively correlated with MMSE scores. PVS was significantly and positively correlated with TMT-A, CRT, and Trails results and negatively correlated with DSST results. Mean global CBF was significantly and positively correlated with TMT-A and CRT results but negatively correlated with DSST results. These findings indicate that high total CSVD burden and focal lesions contribute to cognitive impairment, characterized mainly by attention deficit and executive function. Structural abnormalities in the brain induced by CSVD serve as the core pathological basis of vascular cognitive impairment, involving a cascade of changes such as greater WMH burden, cortical thinning, and the atrophy of gray matter and subcortical nuclei, with a subsequent reduction in their volume. With an increase in total CSVD burden, cerebral microvascular pathology involves a broader anatomical range and causes more severe damage. This directly disrupts the signal conduction capacity of long-range association fibers and secondarily induces structural atrophy and abnormal functional network connectivity across widespread brain regions, thereby decreasing the efficiency of the global cognitive network. The results of a meta-analysis based on the Yeo 7-network model indicated that higher-order cognitive functions, such as executive function, attention, and information processing speed, depend on coordinated interactions among multiple networks, making them more vulnerable to structural damage associated with CSVD. Consequently, impairments in these domains become more evident as the CSVD burden increases (Zhang Z. et al., 2026). This study showed that CSVD severity is correlated with cognitive function from the perspective of clinical burden. This suggests that total CSVD burden can serve as an important neuroimaging biomarker for evaluating the risk of cognitive impairment and monitoring disease progression, thereby providing an objective basis for the early clinical identification of high-risk populations and developing effective intervention strategies.

A large-scale cross-sectional study by Guo et al. revealed that severe OSA remained an independent risk factor for cognitive impairment after adjusting for confounding factors (Guo et al., 2025), whereas in this study, inter-group comparisons showed no significant differences in cognitive test scores among the severe OSA, moderate OSA, and control groups. This discrepancy occurred probably because of the differences in the cognitive assessment tools used. Guo et al. used a single MoCA score to define cognitive impairment, which is more sensitive to mild cognitive impairment, particularly early alterations in executive function and attention. Another reason for this discrepancy might be that cognitive impairment in our cohort was mainly driven by total CSVD burden rather than OSA. Thus, if the total CSVD burden does not considerably increase, even among patients with severe OSA, cognitive impairment associated with OSA may remain at a subclinical stage and therefore fail to show detectable intergroup differences.

Regarding the mechanisms underlying cerebral perfusion and hypoxia, this study found that mean global CBF was significantly and positively correlated with TMT-A scores and negatively correlated with Trails scores. This finding complements the findings reported by Zhang N. et al. (2026): intermittent hypoxia associated with OSA induces a compensatory increase in cerebral blood flow, and chronic hyperperfusion aggravates oxidative stress and neuroinflammation, thereby causing myelin damage and axonal microstructural abnormalities in regions such as the frontal lobe, hippocampus, and cingulate gyrus, ultimately leading to cognitive impairment. A high global CBF serves as a marker of cerebral hypoxic compensation and early injury. Global CBF can serve as a neuroimaging biomarker for evaluating OSA-related neurocognitive impairment.

## 5 Limitations

Although this study yielded valuable clinical findings, it has several limitations that should be addressed in future studies. First, as this study had a cross-sectional design, it could only identify associations among variables, preventing the establishment of causal relationships or the observation of dynamic changes over time; thus, the causal sequence involving OSA, total CSVD burden, and global CBF could not be determined. Second, the small sample size may have led to insufficient statistical power for some analyses, making the detection of subtle differences between variables difficult. Third, although key confounding conditions, such as cerebrovascular and neurological diseases, were excluded, other potential confounders, including chronic comorbidities, unhealthy lifestyles, and long-term medication, were inadequately controlled for, which may have produced residual confounding effects on the results. Fourth, the duration of OSA and its biomarkers, such as oxidative stress, inflammatory cytokines, and vascular endothelial function, were not included. Hence, whether disease duration mediates the association between OSA severity and the development and progression of CSVD could not be determined, and the mechanisms by which OSA induces cerebral microvascular pathology at the molecular level could not be assessed. Fifth, healthy individuals (control) and mild OSA patients were placed in one group. Finally, the location of PVS was not differentiated.

To address the limitations of this study, future research can be improved by implementing the following three aspects. First, the sample size should be increased, and prospective longitudinal studies should be conducted with strict control of potential confounders, such as chronic comorbidities, unhealthy lifestyles, and the administration of medication, to elucidate the pathophysiological mechanisms underlying OSA-induced CSVD from a multidimensional perspective. Second, the duration of OSA should be incorporated, and biomarkers of serum inflammatory cytokines, oxidative stress, and vascular endothelial function should be integrated to assess the effect of disease duration and the regulatory roles of various biomarkers during disease progression. This can provide a theoretical basis and practical support for precision intervention of OSA-related cerebral impairment. Third, mediation and moderation analyses should be performed to examine the mediating effect of total CSVD burden on the relationship between OSA and cognitive impairment. Long-term follow-up is required to record the dynamic changes in BMI, total CSVD burden, global CBF, and cognitive function after interventions such as CPAP therapy and weight reduction, ultimately assessing the causal relationships among these variables.

## 6 Conclusion

By comparing clinical and neuroimaging parameters between controls and patients with different OSA severity levels, the following conclusions were drawn: (1) BMI serves as an important aggravating factor in the development of severe OSA. Patients with severe OSA had considerably higher BMI than those with moderate OSA and controls, whereas the difference between the moderate OSA and control groups was not significant, suggesting that obesity mainly promotes progression to severe disease rather than serving as the initial cause of moderate OSA. (2) A threshold effect was found between total CSVD burden and OSA severity. A significant difference in total CSVD burden was found only between the severe and moderate OSA groups, whereas no difference was found between the moderate OSA and control groups. This suggests that cerebral small vessel injury must continue till the severe OSA stage before significant differences can be detected. (3) Among neuroimaging markers related to CSVD, only PVS showed significant differences among groups, suggesting that PVS is sensitive to the pathological progression of OSA-related CSVD. Additionally, PVS progression could be effectively delayed by standardized CPAP therapy, which indicated that PVS may act as an early biomarker and target for OSA-related cerebral small vessel injuries. (4) OSA was associated with compensatory hyperperfusion and related to CSVD severity. After BMI was controlled, OSA severity remained independently and positively associated with mean global CBF. Additionally, global CBF was significantly associated with PVS and CMBs, suggesting that chronic hypoxia-induced hyperperfusion may be a key mechanism underlying CSVD. (5) Total CSVD burden and mean global CBF were significantly correlated with cognitive function. This finding indicates that neuroimaging characteristics, including total CSVD burden, lacunes, and PVS, strongly influence cognitive function and may serve as neuroimaging biomarkers for clinical evaluation.

## Data Availability

The raw data supporting the conclusions of this article will be made available by the authors, without undue reservation.
