# Supplementary material for: The association between obstructive sleep apnea and total cerebral small vessel disease burden—Neurovascular insights of OSA
Source: Front Neurosci. 2026 Sep 4;20:1916857. doi: 10.3389/fnins.2026.1916857 (PMC13587790; doi:10.3389/fnins.2026.1916857)
Supplement: Supplementary file 2 [file Supplementary_file_2.docx]

Supplementary Materials 2. There was no statistically significant correlation between hypoxia-related indicators and CSVD burden, imaging markers, or cerebral blood flow.

| Variable | Total CSVD burden | lacunes | PVS | CMBs | CBF_Mean |
| --- | --- | --- | --- | --- | --- |
| ODI3% | 0.016/0.843 | -0.019/0.820 | 0.097/0.255 | -0.022/0.774 | 0.003/0.969 |
| ODI4% | 0.013/0.877 | -0.014/0.871 | 0.100/0.245 | -0.033/0.700 | 0.012/0.868 |
| SpO2 Mean | -0.085/0.322 | -0.049/0.586 | -0.173/0.054 | -0.055/0.541 | -0.058/0.432 |
| SpO2 Nadir | -0.071/0.390 | -0.099/0.251 | -0.138/0.111 | 0.001/0.997 | -0.045/0.526 |
| T90 | 0.063/0.444 | 0.073/0.390 | 0.128/0.133 | 0.035/0.682 | 0.051/0.464 |
| RAI | 0.090/0.271 | -0.063/0.458 | 0.122/0.153 | 0.063/0.459 | 0.015/0.832 |
| AHI | 0.062/0.447 | 0.006/0.940 | 0.133/0.117 | 0.005/0.953 | 0.014/0.845 |

Note: r value/P value; ODI3%, Oxygen Desaturation Index 3%; ODI4%, Oxygen Desaturation Index 4%; SpO2 Mean, Mean Peripheral Oxygen Saturation; SpO2 Nadir, Nadir Peripheral Oxygen Saturation; T90, Time Spent Below 90% Oxygen Saturation; RAI, Respiratory Arousal Index; AHI, Apnea-Hypopnea Index
