# Supplementary material for: The association between obstructive sleep apnea and total cerebral small vessel disease burden—Neurovascular insights of OSA
Source: Front Neurosci. 2026 Sep 4;20:1916857. doi: 10.3389/fnins.2026.1916857 (PMC13587790; doi:10.3389/fnins.2026.1916857)
Supplement: Supplementary file 3 [file Supplementary_file_1.docx]

Supplementary Materials 1. Flow diagram of the study.
